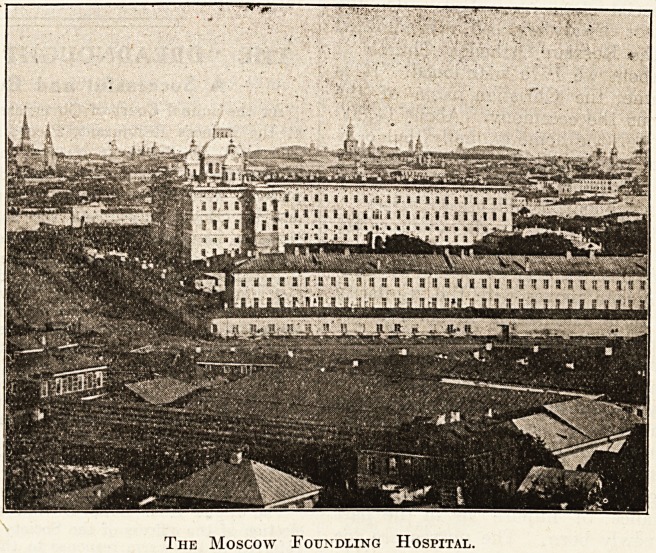# A Famous Foundling Hospital: Vospitatel'nie Dom, Moscow

**Published:** 1916-03-18

**Authors:** Cyril G. E. Bunt


### A FAMOUS FOUNDLING HOSPITAL.

### Vospitatel'nie Dom, Moscow.

By CYRIL G. E. BUNT.

In some respects Russia may be said to be a nation of children. The great majority of those millions who acknowledge the sovereignty of the Great White Tsar are, socially, yet in their adolescence. The stunting effects of centuries of serfdom are not to be eradicated in a single generation, and it may be quite a long time ere it would be sound policy for the Government to relax its paternal methods of administration.

This is not the place in which to dwell upon the extent to which the Russian millions look to the Government as children look to their parent. Nor is it intended to enlarge upon the child-like faith and trust the Russian peasant places in the "Little Father," as he affectionately styles the Tsar. But it would be impossible probably to find a better illustration of the paternal (or shall we say *maternal?*) attitude of the Government of which the "Little Father" is head than the Foundling Hospitals of Moscow and Petrograd. As models of this type of institution they are deservedly famous, yet they show interesting peculiarities which are typically Russian.

The Moscow Foundling Hospital.

All visitors to that most national of all Russian towns—"Our Mother Moscow"—must have been impressed with a sense of wonder on first beholding the huge pile and being told that it is the "Foundling Hospital." It is truly one of the most striking of the many remarkable buildings in the old capital, for it is the largest building in the town, and its colossal white block of four storeys and a basement is impressive principally by reason of its proportions. The front portion covers an area of 301 feet by 112 feet, while the wing (seen extending backwards in the illustration) has an area of 441 feet by 296 feet. All guide-books rejoice in telling that it has 2,228 windows and accommodation for 3,000 souls. It is supported by the Government, whose grant annually amounts to about 1,000,000 roubles, the greater part of which is derived from the sale of playing-cards, in which the Government has a monopoly.

It was founded by Catherine II. in 1764 and fostered by the Empress Maria Feodorovna, wife of Paul I. Before the time of Catherine it had been customary for foundlings to be received at the church windows, by women in the employ of the State. But Catherine, whose passion for building was immense and whose reforms mark her reign as memorable in the annals of united Russia, founded the present institution upon a plan drawn up by General Betski, an eminent philanthropist of the time.

It is not only a place of refuge for the poor outcast little ones of the Empire. It receives its name "Vospitatel'nie Dom" ("House of Education") from the fact that it is also an orphanage for the education of children of past servants of the State. Hence the symbolic appeal of the two groups to be seen before the principal entrance, which are the work of Vitali. They represent Charity and Education respectively.

The two institutions—the Foundling Hospital and the Nicholas Institute—are quite separate in their administration. The latter is in occupation of the first, second, and third floors. It is restricted to the female orphans of indigent servants of the Crown, about 800 of whom are given a liberal education, including languages, physics, elementary mathematics, literature, music, and dancing. The normal course lasts about six years, during which they are not only kept and clothed by the State, but on gaining certain certificates they even enjoy small salaries. They are bound to devote six years to the service of the State as governesses and school teachers. Even as they come from all parts of the vast Empire, so, on the completion of their education, they are sent forth to teach in the State schools from the shores of the Caspian to the White Sea. At their departure from the institution they are provided with clothes and a small sum of money, and at the completion of their six years of teaching, if they do not then secure employment, the institute receives them back. Should any of them get married while yet at the Home they are provided with a complete trousseau—in fact, the State continues its rôle of mother to the last. The fourth floor is given over entirely to the foundlings. There there may be seen as many as a thousand nurses, each with an infant at the breast. On the ground floor is a room wherein is a baptismal font and the reception hall. Here on all days, and at any hour, the poor infants whose parents have abandoned them are received in a true spirit of Christian charity. In Russia there is no such thing as a child being left at the door of the institution, as might happen elsewhere. They are taken openly by some person interested in them, no question being asked as to who are its parents, nor, indeed, any question except whether it has been baptised, and, if so, what its Christian name may be.

Each child is registered on entrance and a number is assigned to it, and the person who brought the infant may reclaim it by this number, if so disposed, any time up to the age of ten. After registration the child is undressed, washed, and clothed in the special clothes supplied. On the following day, if not already baptised, it is admitted into the fold of the Russian Orthodox Church at the font in the room we have mentioned. It is given, as a surname, the Christian name of the priest who performs the ceremony. About 14,000 children pass through the home annually, but only the weakly remain any length of time. The strong and healthy are vaccinated, and after four weeks are sent into the neighbouring villages to nurse.

The cleanliness of the great wards is admirable. The domestic and sanitary arrangements are everything that could be desired, and the whole machinery of the huge *crèche* moves with the regularity of clockwork. It is astonishing to observe the great neatness prevailing in the dormitories, considering the continual change of nurses that is ever taking place. Each has, close to her own bed, a cradle for her charge, and near-by a bath of copper —in fact, all necessary adjuncts to perfectly hygienic conditions. All dirty linen is conveyed by an ingenious arrangement direct to the laundry situated on the ground floor, thus reducing to a minimum any chance of impure air in the part occupied by the newly born. The nurses are for the most part country women, who, being capable of acting as foster-mothers to the waifs, are attracted by the good wages paid by the State. The continual change of nurses above referred to is a peculiar result of the system of coping with the immense number of infants that pass through the institution each year. For after four weeks in the home they are sent with their foster-mothers to the villages to which the latter belong; and the State allows a definite sum for their maintenance. It is said that some mothers have taken unfair advantage of this arrangement by getting their own child admitted as a foundling, and then, being already on the list of nurses, actually securing that it should be boarded out with them at the expense of the State.

The children are reared thus in the country until they attain the age of five years. They then return to the Home and are well grounded in the rudiments of education. The greater number of the boys (who are all, of course, liable for military service) return to the land as agricultural labourers. A great number of the girls become either nurses at the hospital or midwives, for both of which professions there are special schools attached to the institution. They stay at the Home until they are eighteen, when they are given their freedom, thirty roubles in money, and two suits of clothes.

There is also a lying-in hospital attached to the Home, which is open not only to poor mothers, but also to those in a more unfortunate state even than poverty. For the latter there are secret wards, in which each patient is known only by a number, and sometimes as many as 2,000 unfortunates are given admittance in a year. The church of the establishment is very fine, and is situated in the principal block, seen in the illustration surmounted by three graceful cupolas. It has an artistically carved dome supported by columns of rose-coloured marble.

It is worthy of note that in the campaign of 1812 the building was used by Napoleon as a military hospital.

## Figures and Tables

**Figure f1:**